# A Dynamic Surface Metal‐Organic Framework Electrocatalyst: Potential‐Driven Activation of Atomically Defined Au Sites for the Hydrogen Evolution Reaction

**DOI:** 10.1002/smll.75601

**Published:** 2026-09-12

**Authors:** Paulina Wira, Leonardo Cielo, Zisheng Zhang, Mattia Cattelan, Rafał Lewandków, Dennis Pedersoli, Fujia Liu, Boyu Qie, Felix. R. Fischer, Francesco Sedona, Cristina Tubaro, Tomasz Kosmala, Stefano Agnoli

**Affiliations:** ^1^ Institute of Experimental Physics University of Wrocław Wroclaw Poland; ^2^ Department of Chemical Sciences and INSTM Research Unit University of Padua Padua Italy; ^3^ Department of Chemical Engineering Stanford University Stanford USA; ^4^ SUNCAT Center for Interface Science and Catalysis SLAC National Accelerator Laboratory Menlo Park USA; ^5^ Department of Chemistry University of California Berkeley California USA; ^6^ Kavli Energy Nanosciences Institute University of California Berkeley and the Lawrence Berkeley National Laboratory Berkeley California USA; ^7^ Materials Sciences Division Lawrence Berkeley National Laboratory Berkeley California USA; ^8^ Bakar Institute of Digital Materials For the Planet Division of Computing Data Science and Society University of California Berkeley Berkeley California USA

**Keywords:** hydrogen evolution reaction, nitrogen heterocyclic carbenes, operando scanning probe microscopy, surface metal organic frameworks

## Abstract

Surface metal‐organic frameworks (SMOFs) are promising platforms for catalysis, yet their structural response to electrochemical bias is often overlooked. We report the potential‐driven activation of an N‐heterocyclic carbene (NHC)‐based SMOF on Au(111) for the hydrogen evolution reaction (HER). *Operando* electrochemical scanning tunneling microscopy (EC‐STM) reveals that the framework, initially “frozen” in a strongly interconnected 1D chain morphology at open circuit potential, transitions into a dynamic phase under cathodic bias. This reconfiguration involves a dynamic cycle of metal abstraction and release, generating a high density of undercoordinated sites. DFT calculations confirm that while internal bridging Au nodes are inert (ΔG_H*_ = 0.99 eV), the potential‐induced decoupling of the chains creates terminal Au nodes and lattice vacancies with near‐thermoneutral adsorption energies of 0.05 and 0.07 eV, respectively. This study demonstrates that superior HER performance originates from the SMOF's ability to dynamically transform into a fluxional active state, providing a strategy for designing adaptive interfacial catalysts.

## Graphical abstract

Operando EC‐STM reveals that an NHC‐based SMOF on Au(111), initially frozen in interconnected 1D chains, dynamically reconfigures under cathodic bias into a fluxional structure bearing undercoordinated Au sites while abstracting Au atoms from the support. DFT calculations identify these undercoordinated sites and Au vacancies as the active sites for HER, unveiling a bias‐activated pathway for designing adaptive, dynamically self‐optimizing catalysts.

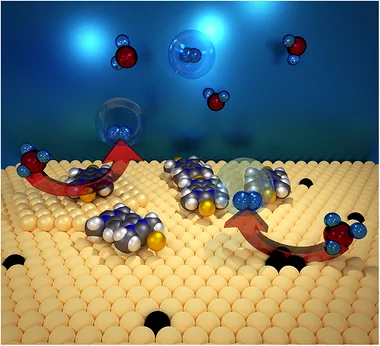

## 1 Introduction

The development of surface metal‐organic frameworks (SMOFs) has opened a new frontier in interfacial science, offering a bridge between the predictability of molecular chemistry and the robustness of heterogeneous catalysis [1]. Unlike 3D MOFs, 2D SMOFs are constrained to a surface, allowing for direct electronic and chemical coupling with the support [2, 3, 4]. The true power of these systems lies in their modular architecture: by selecting specific organic “linkers” and metal “nodes”, the geometric and electronic environment of the SMOF can be tuned with atomic precision [5, 6].

Among the various building blocks used to assemble these frameworks, N‐heterocyclic carbenes (NHCs) represent a paradigm shift. While traditional thiol‐based monolayers often act as passive spacers, NHCs function as active structural directors. Their exceptional σ‐donating ability allows them to covalently bond to the metal lattice and, crucially, to abstract metal atoms from the substrate [7, 8, 9]. This process transforms a flat, relatively inert surface like Au(111) into a highly organized, carbene‐stabilized SMOF. This surface‐derived framework is unique because the metal nodes are not added from an external precursor but are extracted directly from the electrode itself. By isolating individual gold atoms from the bulk lattice and stabilizing them within an organic matrix, the NHCs effectively create a 2D array of atomically defined catalytic centers. This ensures unhindered interfacial charge transfer and provides a unique platform to study the behavior of isolated Au sites in a heterogeneous environment.

Despite the potential of SMOFs for energy applications, their behavior under operating conditions remains a subject of intense debate. While studies on the oxygen evolution or reduction reactions frequently report the *in situ* transformation of frameworks into metal oxides or amorphous phases [10, 11, 12, 13], the *operando* structural evolution of these interfaces during the Hydrogen Evolution Reaction (HER) is less investigated and poorly understood [6, 14]. On a broader perspective, the question is whether the organic linkers can maintain a stable yet active coordination environment under complex conditions, which may entail solvent, reactive chemical species, and local electric field [15, 16]. A central challenge is understanding the dynamic nature of these frameworks—how they breathe and restructure under the action of chemical stimuli (e.g., pH) or the application of an external potential [14]. In this work, we use electrochemical scanning tunneling microscopy (EC‐STM) to provide a microscopic insight into the *operando* dynamics of an NHC‐based SMOF in electrolytic media.

We demonstrate that the properties of this SMOF are inherently potential‐dependent. In the double‐layer stability region, the framework exists in a resting state, where the carbene‐Au(I) units are locked into interconnected linear chains that are spatially and conformationally fixed, as documented in previous work under ultra‐high vacuum (UHV) conditions [8]. However, as the system approaches the reducing potentials required for HER, the SMOF undergoes a dramatic activation. We observe the onset of molecular dynamics, where the framework units disconnect from each other and gain lateral mobility, eventually behaving like a 2D gas [17]. This transition is accompanied by a significant modification of the Au support: Au atoms are dynamically extracted from the topmost layer and redistributed on the surface, producing a landscape of both atom vacancies and new ad‐islands.

By combining these real‐time observations with density functional theory (DFT), we pinpoint the unique property of this SMOF that triggers HER activity: the presence of a terminal Au(I) active site. Bonded on one side to the carbene, this undercoordinated gold node possesses a d‐band structure optimally tuned for an almost thermoneutral Gibbs free energy for hydrogen adsorption (ΔG_H*_ ≈ 0).

Our results show that the carbene does not merely stabilize the gold; it redefines the metal's chemical identity within the framework. While SMOFs are often conceptualized as static crystalline lattices, our results suggest they behave as non‐equilibrium catalysts where the true working state only emerges through a potential‐driven structural reconfiguration. This system exemplifies a dynamic catalytic surface, characterized by a fluxional cycle of metal abstraction and release that operates far from thermodynamic equilibrium, effectively bridging the gap between rigid model surfaces and dynamic industrial catalysts [18].

## 2 Results and Discussion

### 2.1 Structural Baseline: Pre‐Electrochemical Characterization

In our previous studies, we established the structural and electronic foundations of the NHC‐Au(111) interface [7, 8]. Through a combination of scanning tunneling microscopy (STM) and X‐ray photoelectron spectroscopy (XPS), we demonstrated that the carbene precursor 1,3,5,7‐tetramethyl‐3,7‐dihydrobenzo[1,2‐d:4,5d’]diimidazole‐1,5‐diium bis(tetrafluoroborate), hereafter named (bis‐NHC^Me^)·2HBF_4_ can assemble on the Au(111) surface forming extended SMOF characterized by interconnected linear chains of NHC‐Au^δ+^‐NHC units. The resulting SMOF is a surface‐confined, flat‐lying NHC–Au–NHC coordination network, in which the Au atoms extracted from the substrate bridge neighboring carbene units.

High‐resolution STM imaging confirms the formation of long‐range ordered, 1D arrays where the Au nodes appear as round bright protrusions, characterized by spacing of ∼1 nm [7]. These chains are formed via the spontaneous abstraction of Au atoms from the substrate, which are then site‐isolated by the bis‐carbene molecules to create a periodic array of atomically defined Au coordination nodes. Notably, the morphology of the Au support is not affected by the formation of these nanoarrays.

Previous characterization with synchrotron radiation XPS demonstrated that the electronic state of these gold nodes is consistent with a partially oxidized Au. Moreover, the SMOF chains exhibit exceptional thermal and chemical resilience. Thermal stability is maintained up to 200°C, and *ex situ* characterization (Figure S1) demonstrates that the 1D morphology remains invariant even after 24 h of exposure to ambient conditions. In this pre‐catalyst state, the SMOF is conformationally fixed and acts as a passivating layer, with the bridging gold nodes largely inaccessible for reaction, a resting configuration that serves as the necessary starting point for our *operando* investigations.

### 2.2 Operando Structural Evolution and Dynamic Activation

Encouraged by the good stability of the NHC–Au linkage, we extended our investigation to harsher, application‐relevant regimes to evaluate the potential of these isolated metal nodes as stable electrocatalytic sites for the HER. To this end, we employed *operando* EC‐STM to investigate the structural integrity of the SMOF on Au(111) across a wide range of pH conditions, specifically in 0.1 M HClO_4_ (acidic), 0.1 M KHCO_3_ (pH 8.3), and 0.1 M KOH (alkaline) environments within the double‐layer stability window (Figure 1).

**FIGURE 1:**
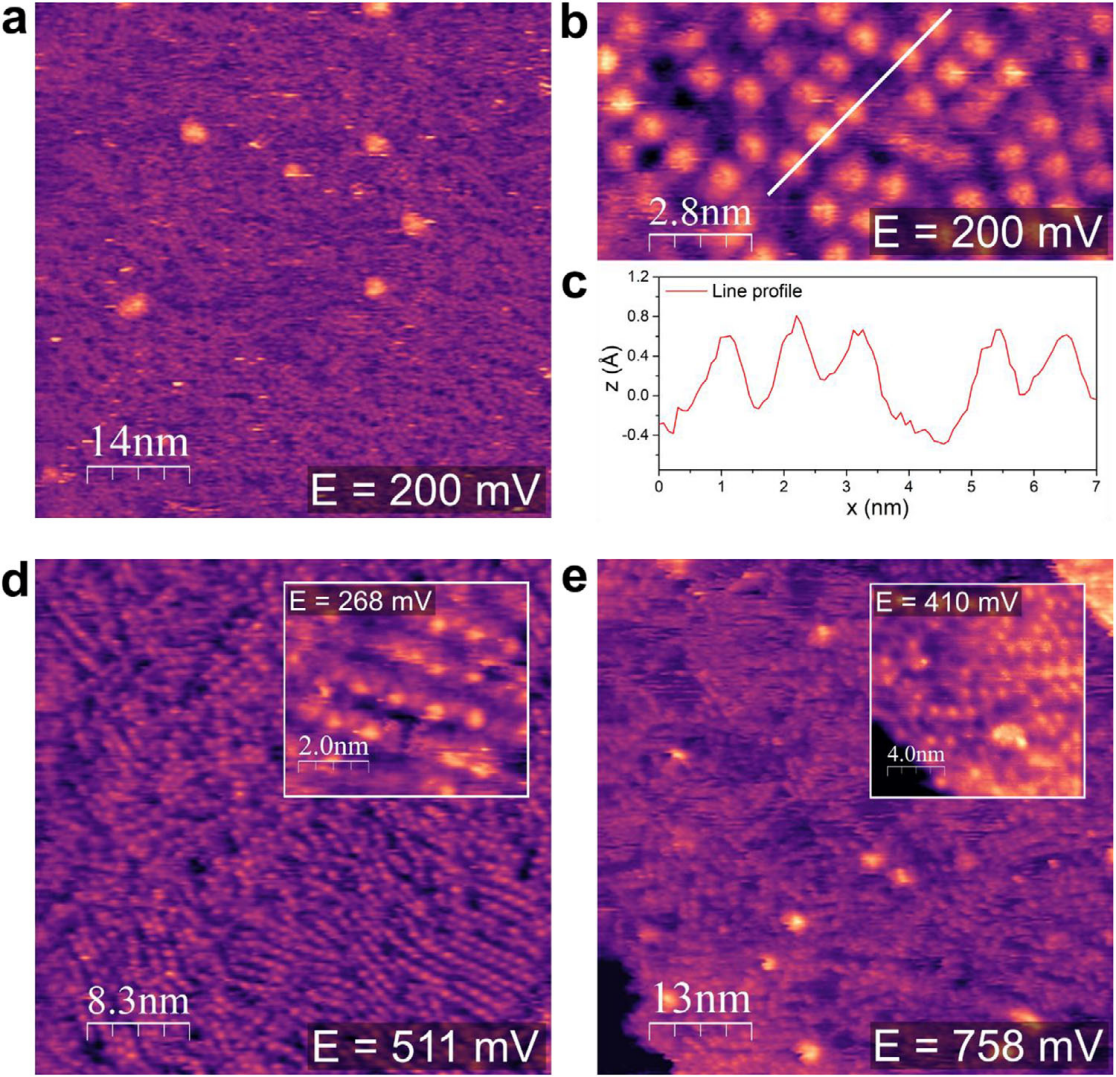
In situ EC‐STM images of the SMOF on Au(111) recorded in the double‐layer regime after several hours of exposure to electrolytes of different pH. (a) Large‐scale image in 0.1 M HClO_4_ at 200 mV. (b) High‐resolution image in 0.1 M HClO_4_. (c) Corresponding apparent‐height profile. (d) Large‐scale image in 0.1 M KHCO_3_ with a high‐resolution inset. (e) Large‐scale image in 0.1 M KOH with a high‐resolution inset. The characteristic 1D chain structure is preserved in all three electrolytes. Tunneling conditions: (a) U_b_ = 81 mV, I_t_ = 0.48 nA, E = 200 mV; (b) U_b_ = −38 mV, I_t_ = 0.48 nA, E = 200 mV; (d) U_b_ = −245 mV, I_t_ = 1 nA, E = 511 mV; inset: U_b_ = −104 mV, I_t_ = 1 nA, E = 268 mV; (e) U_b_ = −205 mV, I_t_ = 1 nA, E = 758 mV; inset: U_b_ = 153 mV, I_t_ = 1 nA, E = 410 mV.

In all media, we achieved molecular resolution, allowing us to monitor the surface evolution over several hours. Our observations confirmed the persistence of the same 1D chains documented under UHV conditions, maintaining identical Au–Au node spacing (1 nm) and similar inter‐row separation (1.0–1.4 nm). Notably, even after several hours, no appreciable degradation in surface coverage or change of geometric features was observed, demonstrating the remarkable chemical resilience of the carbene‐gold bond against protonation or hydroxyl attack.

Comparing the results of SMOFs prepared via liquid‐phase deposition (Figure 1a,b) with those synthesized via UHV methods (Figure 1d,e), we observe no significant differences in their structural and morphological properties [8]. Generally, liquid‐phase synthesized samples exhibit a lower degree of long‐range order and shorter chain lengths, which is consistent with previous reports [8, 19]; however, their stability in electrochemical environments is comparable to that of UHV‐prepared surfaces. Thus, although chain length, mesoscopic order, and crystallographic alignment with the underlying substrate can vary with the preparation route, the local 1D NHC–Au coordination motif and the intrinsic stability of the NHC–Au^δ+^ units are preserved in all electrolytes. This suggests that while the mesoscopic order depends on the preparation method, the intrinsic stability of the NHC–Au^δ+^ units is a defining feature of the surface‐derived framework regardless of the synthetic pathway.

Finally, the electrocatalytic activity toward HER was evaluated in acidic conditions (0.1 M HClO_4_, Figure 2). The NHC‐based SMOF shows a clear enhancement in catalytic activity compared to the pristine Au(111) surface. This is evidenced by a positive shift in the onset potential from −163 mV for Au(111) to −106 mV for the NHC‐decorated surface, as well as a reduced overpotential of −125 mV required to reach a current density of 500 µA cm^−2^.

**FIGURE 2:**
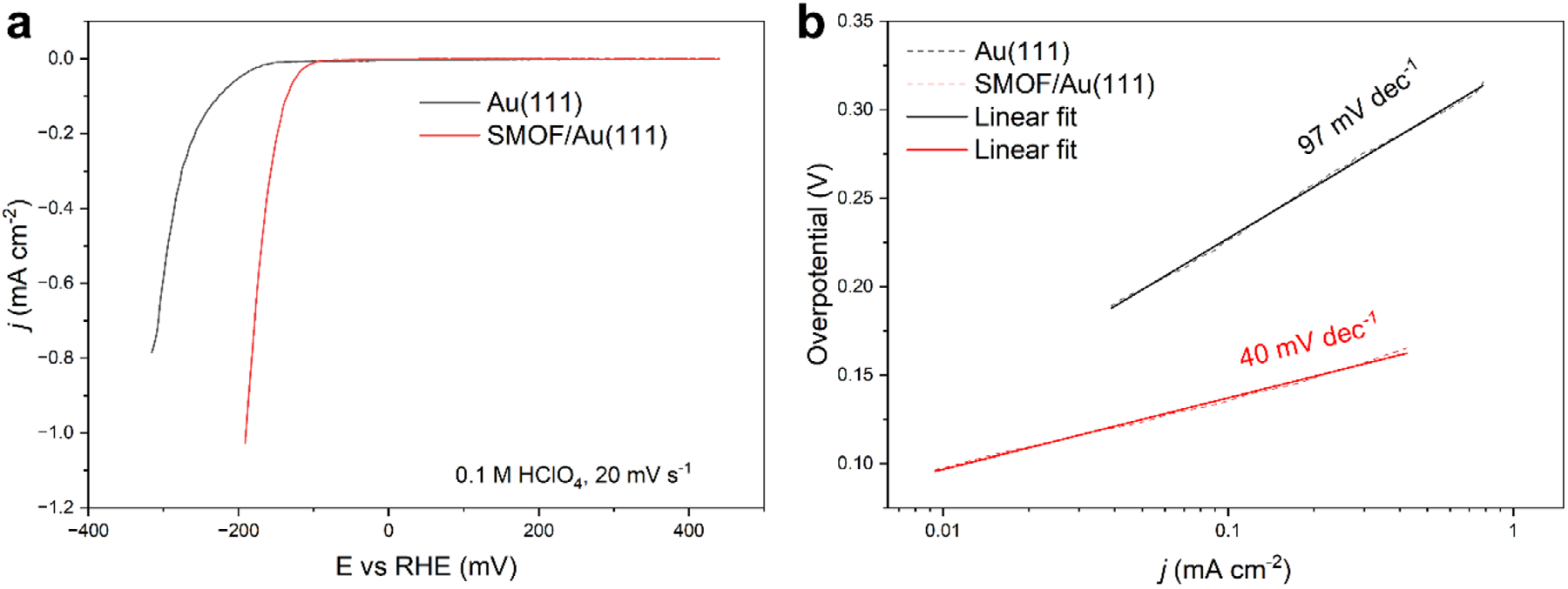
HER activity of the SMOF‐decorated and bare Au(111) surfaces. (a) LSVs in Ar‐saturated 0.1 M HClO_4_, scan rate 20 mV s^−1^. (b) Tafel plots.

Tafel analysis further suggests a change in reaction pathway for the HER (Figure 2b). On Au(111), the Tafel slope is ∼96 mV dec^−1^, indicating mainly a Volmer‐limited mechanism, in line with the typical weak hydrogen adsorption on gold surfaces. In contrast, the SMOF/Au(111) surface exhibits a lower Tafel slope of ∼40 mV dec^−1^, which indicates a modification of the rate‐determining step, likely enabling a competitive Heyrovsky (electrochemical desorption) process, as a consequence of the presence of catalytic sites with improved hydrogen binding and faster interfacial charge transfer kinetics, although mixed kinetic control cannot be excluded.

The potential‐dependent behavior of the SMOF was investigated using *operando* EC‐STM to bridge the gap between static structural models and active electrocatalysis. In the capacitive potential window, the framework remains in an almost frozen state, characterized by relatively rigid, permanently interconnected 1D chains (Figure 1a). In this regime, the Au nodes are resolved as distinct, immobile bright features with an apparent corrugation of about 1–1.5 Å depending on the tunneling bias and show the same degree of order and geometric features documented under UHV conditions.

As the electrode potential is swept toward increasingly cathodic (reducing) potential, a dramatic transition in the framework's stability and mobility is observed, as reported in Figure 3 and Movie S1. The linear arrays begin to exhibit significant lateral fluctuations, adopting increased conformational fluxionality. Potentiodynamic EC‐STM imaging reveals that the Au nodes gain substantial lateral mobility, disconnecting from their neighbors and diffusing across the terrace. It must be noted that, at HER‐relevant potentials (< −0.15 V), the SMOF units are too mobile to be resolved as a static molecular structure on the timescale of STM acquisition. Importantly, stable STM imaging of the surface is maintained; however, the rapid diffusion and structural fluxionality of the framework give rise to characteristic streaks and spikes, which reflect transient tip–molecule interactions during scanning.

**FIGURE 3:**
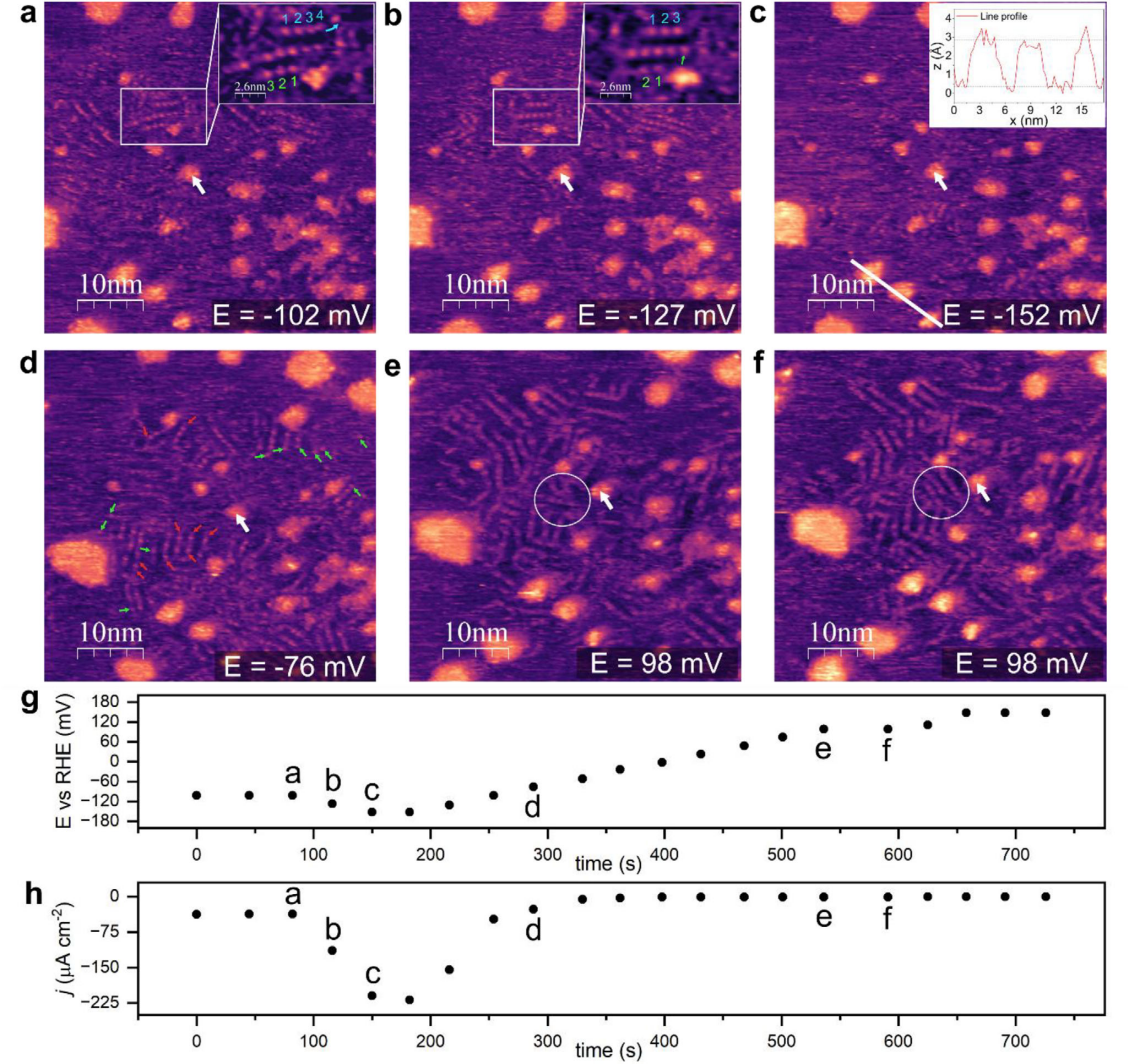
*Operando* EC‐STM potentiodynamic sequence showing the evolution of the SMOF/Au(111). Panels (a–f) are selected EC‐STM frames extracted from Movie S1. Insets in (a) and (b) highlight local fragmentation and restructuring of SMOF across the surface; some transiently trapped Au atoms are released, as indicated by the blue arrow in the inset of (a). The inset in (c) shows a height profile along the line marked in panel (c), with an apparent height of ∼2.4 Å, consistent with monolayer Au ad‐islands. In (d), the green and red arrows indicate 1D chains terminated by a terminal Au atom and an NHC molecule, respectively. Panels (g) and (h) show the corresponding electrode potential and current density vs time, respectively, with the acquisition points of panels (a–f) indicated together with the corresponding E and *j* values. In panels (a–f), the white arrow indicates the same Au ad‐island in successive frames. Tunneling conditions: (a) U_b_ = −260 mV, I_t_ = 1 nA, E = −102 mV, *j* = −36.9 µA cm^−2^; (b) U_b_ = −260 mV, I_t_ = 1 nA, E = −127 mV, *j* = −114.1 µA cm^−2^; (c) U_b_ = −260 mV, I_t_ = 1 nA, E = −152 mV, *j* = −209.7 µA cm^−2^; (d) U_b_ = −300 mV, I_t_ = 1 nA, E = −76 mV, *j* = −26.5 µA cm^−2^; (e) U_b_ = −300 mV, I_t_ = 1 nA, E = 98 mV, *j* = −0.69 µA cm^−2^; f) U_b_ = −300 mV, I_t_ = 1 nA, E = 98 mV, *j* = −0.67 µA cm^−2^.

The set of images in Figure 3 and Movie S1 capture the dynamic reorganization of densely packed 1D molecular arrays. As indicated by the white ellipses in Figure 3e,f, several arrays exhibit dynamic connectivity and conformational rearrangements between consecutive images. The insets in Figure 3a,b further detail this behavior, highlighting specific regions where chains shorten due to the loss of terminal Au nodes, accompanied by the stochastic displacement of isolated bright features. This structural transition occurs over a 55 s interval at a constant potential of 98 mV, underscoring the inherent mobility and dynamic nature of the chain architecture under these electrochemical conditions. Importantly, this dynamic behavior also persists under constant HER polarization. Four consecutive EC‐STM images recorded at −121 mV vs RHE, corresponding to a current density of approximately −86 µA cm^−^^2^, show continued diffusion and reorganization of the molecular framework (Figure S2). Thus, the fluxional state is maintained during sustained HER operation and does not arise solely from continuous changes in the applied potential.

The potential dependence and partial reversibility of this mobility are further demonstrated by potential‐step experiments.

When the electrode potential is rapidly stepped from catalytic conditions (e.g. −121 mV) to a more positive value within the capacitive region (e.g. 727 mV), the SMOFs undergo a distinct transition. While initially difficult to resolve due to rapid diffusion at catalytic potentials, they stabilize within seconds of the anodic step, appearing as a coherent and relatively static surface network (Figure S3).

Cathodic polarization also drives restructuring of the underlying Au(111) substrate. After stepping the potential to HER onset, we observe the nucleation and growth of monolayer Au ad‐islands across the surface (Figure S4). These islands typically consist of a single monolayer (apparent corrugation 2.4 Å, see line profile in Figure S4) and adopt well‐defined triangular or hexagonal shapes. The uniform alignment of their edges indicates a simple (1 × 1) epitaxy with the underlying Au(111) lattice (Figures S3 and S4). Depending on the local substrate morphology, such as the proximity to step edges and the surface molecular density, the ad‐islands can reach lateral dimensions of 15–25 nm.

Quantitative analysis of the morphology changes during the constant‐potential EC‐STM experiment at −105 mV vs RHE, summarized in Table S1, reveals a distinct two‐step growth mechanism: rapid initial nucleation followed by lateral coarsening. This sequence is evidenced by a marked increase in island number during the potential hold, coupled with the initial decrease and subsequent increase in mean island area. This complementary observation demonstrates that sustained cathodic polarization drives not only molecular diffusion within the SMOF but also progressive redistribution of substrate Au. It is worth noting that control experiments of pristine Au(111) under identical acidic conditions and potential window reveal no changes in surface morphology (Figure S5), consistent with previous reports [20].

Therefore, the body of our experimental data suggests a reversible cycle of metal abstraction and release mediated by the molecular framework: the carbene units actively extract Au atoms from the topmost Au(111) layer to assemble the SMOF chains. This dynamic process is experimentally supported by the frequent observation of vacancy islands and shallow pits, appearing as transient dark regions interspersed within the molecular network, which serve as the physical footprint of the metal extraction process (Figure S6). Subsequently, if the surface is polarized toward cathodic potentials, the chains become highly mobile, fragmenting and restructuring across the surface, and some of these captured Au atoms are released, migrating across the terrace to nucleate into the observed ad‐islands. This potential‐driven mass transport effectively refreshes the interface, maintaining a high density of the undercoordinated terminal Au sites (Figure 3d and Figure S7) and surface vacancies that our DFT calculations identify as the primary active centers for HER (*vide infra*).

Even if highly mobile in reducing conditions, the 1D chains are otherwise strongly interacting and stable on the surface, and multiple cyclic voltammetry scans can be performed without significant loss of the surface coverage and of the catalytic activity (Figure S8). In addition, the surface morphology remains largely stable after the first few reductive cycles that trigger the nucleation of the initial Au ad‐islands. As clearly observed, the gold adatom‐islands undergo a dynamic evolution, expanding during the cathodic sweep (Figure S4) and contracting during the anodic sweep (Figure S3). Despite these cyclical potential driven changes, both the population density and spatial distribution of the islands eventually converge to a stationary state. This stabilization is likely mediated by the NHC chains, which maintain a strong preferential anchoring at the island edges even under reducing potentials, effectively “pinning” the morphology in place. The effective passivation of step edges by the carbenes also suggests that these sites are unlikely to be the primary source of catalytic activity.

*Ex situ* XPS analysis indicates that, even after several hours of catalytic operation, the molecular layer undergoes only minimal changes in its chemical composition.

While the C 1s region is difficult to interpret due to unavoidable adventitious carbon contamination, the N 1s region provides more informative insights. Specifically, the N 1s core level shows an increase in full width at half maximum (FWHM) along with the appearance of a low‐intensity shoulder on the higher binding energy side of the main peak (Figure S9).

Multipeak fitting reveals that the main peak at 400.5 eV, which is characteristic of Au–carbene bond formation, remains the dominant component after electrocatalysis, decreasing only from 77% to 63% of the total signal. The shoulder resolved at higher binding energy (401.5 eV) is associated, in the as‐prepared SMOF, with the diimidazolium precursor still bound to the BF_4_^−^ counterion, accounting for only 6% of the signal, whereas in the sample electrocatalysis, this component is attributed to protonated amine species, likely formed through partial ring opening of the carbene under aqueous electrocatalytic conditions, [21, 22] and accounting for 16% of the overall spectrum intensity.

A further minor component appears at 399.0 eV and has previously been attributed to generic structural defects, also observed under harsh conditions such as excessive annealing; it accounts for about 19% of the signal in both the as‐prepared and post‐EC samples [8].

### 2.3 DFT Calculations

To gain molecular level insights into the origin of the enhanced electrocatalytic HER activity at the NHC‐Au(111) interface, we performed DFT calculations to evaluate hydrogen adsorption thermodynamics on various candidate active sites (see Figures S10 and S11 for the space‐filling structural models). The Gibbs free energy of hydrogen adsorption, ΔG_H*_ is used as the descriptor for HER activity (see Figure 4 and Table 1), where a Sabatier‐optimal catalyst would have a near‐zero ΔG_H*_ to strike a balance between proton reduction and product release [23].

**FIGURE 4:**
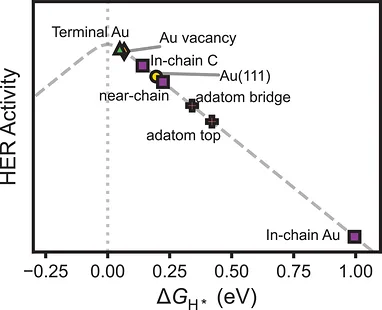
HER activity volcano plot of the candidate active sites. Each marker represents a *H configuration, color‐ and marker‐grouped by the underlying structural motif. Dashed curve marks the ideal activity‐ΔG_H*_ relationship, and the dotted vertical line marks the Sabatier optimum.

**TABLE 1:** Calculated hydrogen adsorption free energies, ΔG_H*_, and simulated exchange current normalized with respect to an idealized optimal catalyst log(*i*_0, norm_), as a descriptor of the HER activity for different structural units of the SMOF/Au(111) interface.

Structural unit	ΔG_H*_ (eV)	*log(i_0, norm_) (a.u.)*
Au(111)	0.196	−3.2876
Au vacancy	0.066	−1.1798
adatom bridge	0.341	−5.7212
adatom top	0.420	−7.0615
Terminal Au	0.049	−0.9405
In‐chain Au	0.995	−16.7206
In‐chain C	0.141	−2.368
near‐chain	0.223	−3.7445

The pristine Au(111) surface has ΔG_H*_ = +0.20 eV, suggesting that H binds too weakly on it, i.e., under‐binding energetics. In the vicinity of the carbene methyl wingtips, H binding on pristine Au(111) is slightly weakened to ΔG_H*_ = +0.22 eV, suggesting that the molecular edges have negligible non‐covalent lateral interactions with the reaction intermediate and are largely spectators in the electrochemical process.

As the SMOF chains migrate, break, and reconfigure over the surface, vacancies and adatoms are *in situ* generated and may serve as HER sites. Au vacancies show significantly stronger H binding of ΔG_H*_ = +0.07 eV; whereas Au adatoms exhibit even weaker H binding (ΔG_H*_ = +0.34 eV) than the pristine Au(111).

When the Au adatom is bonded to one NHC ligand, which can be present at the chain terminations or form during chain breakage, its H* binding is strengthened to ΔG_H*_ = +0.05 eV, offering the highest HER activity among all sites considered in this study. On the contrary, the in‐chain Au nodes bridging two NHC units within the intact framework exhibit markedly weakened H binding of ΔG_H*_ = +0.99 eV, as the linear NHC‐Au‐NHC motif is too rigid and coordinatively saturated. In other words, the static 1D‐SMOF within the double‐layer region is inert *per se*, and it can only be activated by potential‐driven into a dynamic self‐reconfiguring equilibrium where a persistent population of terminal Au sites dictates the HER activity.

Besides Au, we also tested H* adsorption on the carbenic center, which is the only feasible non‐metal adsorption site in this system. Its H* binding strength is at ΔG_H*_ = +0.14 eV, which should offer decent HER activity. However, the adsorption requires the node‐linker motif to geometrically distort into a near‐breakage configuration, with the C‐Au‐C angle bending from 156° to 93° and the C‐Au distance elongating from 1.96 to 2.50 Å. Such drastic geometric distortions would be kinetically unfavorable and have rare occurrence despite their thermodynamic accessibility. This kinetic hinderance is evidenced by the robustness and resilience of the SMOF structure in *operando* EC‐STM experiments, remaining intact over several hours of sustained cathodic bias. Consequently, while the carbenic sites are hypothetically ‘active’ in a thermodynamic sense, their chemical role is kinetically limited.

To conclude, the electrochemical activation of SMOF involves a dynamic equilibrium between the intact and extended spectator chains and a fluctuating ensemble enriched in highly HER‐active terminal sites (ΔG_H*_ = +0.05 eV) and surface vacancies (ΔG_H*_ = +0.07 eV). The improved electrocatalytic activity arises from the low‐coordination nature of the Au sites, which frees up valence for strengthened adsorption and pushes Au's under‐binding energetics up toward the volcano apex. Note that similar effects have been observed in heterogeneous gold catalysis where *in situ* generated defects or clusters dictate the catalytic turnover [24, 25]. However, the resulting active sites are thermo‐populated without fine control of the size and spatial distribution, and the majority of the species would stay or eventually sinter into the inert crystalline phase [18]. In contrast, this work represents a distinct approach where we leverage organic surface chemistry to construct a robust dynamic ensemble of atomically isolated low‐coordinated active centers, offering both superior electrocatalytic performance and molecular‐precise customizability.

## 3 Conclusions

In summary, we have demonstrated that the NHC‐based SMOF exhibits a pronounced potential‐dependent structural response under HER conditions, evolving from a static, frozen architecture in the capacitive region into a highly dynamic, adaptive interface under cathodic polarization, as summarized in Scheme 1. By combining *operando* EC‐STM with DFT calculations, we provide direct experimental evidence that this transition is accompanied by a continuous cycle of metal abstraction and release, generating a high density of undercoordinated gold sites and surface vacancies that act as the true catalytic centers.

**SCHEME 1:**
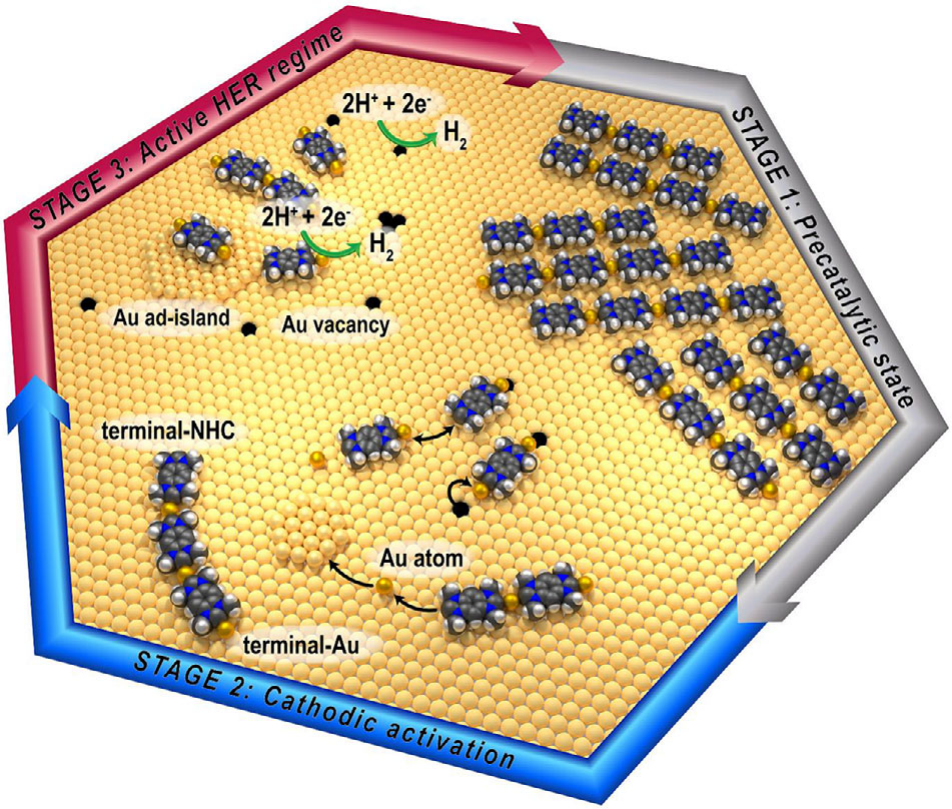
Potential‐controlled activation pathway of the SMOF/Au(111) interface. The ordered pre‐catalytic framework forms through molecular coordination to Au atoms abstracted from the Au(111) surface (stage 1). Cathodic polarization converts this static‐looking architecture into a dynamic molecular–metal ensemble, in which framework mobility, Au release, and surface diffusion drive the formation of Au adatom islands (stage 2). The HER‐active state is therefore generated under operating conditions and is characterized by a continuously restructured interface enriched in undercoordinated terminal Au sites and Au vacancies (stage 3).

This work represents one of the few experimental examples of *operando* characterization of a SMOF under electrocatalytic turnover, and, more broadly, one of the rare cases where dynamic restructuring of a surface‐confined framework is directly visualized in real time during HER. These findings challenge the conventional view of SMOFs as static architectures and instead highlight their inherently fluxional nature under electrochemical conditions.

Importantly, the implications of this study extend beyond electrocatalysis. The observed behavior points to a more complex and dynamic interaction between carbene ligands and metal surfaces than previously recognized. In particular, the ability of NHCs to reversibly extract, stabilize, and redistribute metal atoms under bias suggests that carbene‐functionalized metal interfaces may exhibit similarly adaptive responses in a wide range of electrochemical environments. This has potential consequences not only for catalytic processes, but also for the design of advanced materials in sensing, electrochemical energy storage, and nanoscale interface engineering, where stability and functionality are often governed by subtle, dynamic surface phenomena.

Overall, our results establish NHC‐based SMOFs as a unique platform for the study and design of adaptive, atomically defined catalytic interfaces, and open new directions toward exploiting the dynamic behavior of carbene–metal systems in complex, working environments.

## 4 Experimental Section

### 4.1 Synthesis protocols

#### 4.1.1 UHV‐Synthesis

The NHC‐based SMOFs were deposited by physical vapor deposition (PVD) under ultra‐high vacuum (UHV, 2 × 10^−^^10^ mbar) by heating a crucible containing the diimidazolium precursor to 498 K, producing an estimated flux of approximately 0.033 monolayer min^−^^1^. During deposition, the Au(111) single crystal was kept at room temperature.

Prior to deposition, the substrate was cleaned by repeated cycles of Ar^+^ sputtering (sputtering current I_s__p_u_t_ ≈ 3 mA, Ar pressure p_a__r_ = 1.7 × 10^−^^8^ mbar, acceleration voltage 0.5 kV) followed by annealing at 723 K under pressures below 1 × 10^−^^5^ mbar, followed by annealing at 900 K under UHV conditions.

Liquid‐phase synthesis: A single‐crystal Au(111) electrode (hat‐shaped geometry, surface diameter 8 mm, thickness 2 mm, miscut < 0.5°, roughness < 0.03 µm; MaTeck, Jülich, Germany) was flame‐annealed to obtain a clean and atomically smooth surface. The crystal was placed on a ceramic (Ceran) plate and heated with a butane/oxygen flame for approximately 3 min after reaching a faint red glow (≈600°C–700°C). It was then cooled to room temperature (≈6 min) under Ar, reheated to a red glow, and cooled again for approximately 15 min. The sample was subsequently transferred into a tightly sealed Teflon container filled with 30 mL of a 50 µM ethanolic solution of the diimidazolium salt and kept in an oven at 70°C for approximately 24 h. The sample was removed from the solution, thoroughly rinsed with ethanol, and dried under a stream of Ar.

### 4.2 Characterization

#### 4.2.1 Electrochemical and Operando EC‐STM Measurements

Electrochemical and *operando* EC‐STM measurements were carried out using a home‐built instrument operated in the constant‐current mode, as described in detail by Wilms et al. [26]. All electrolytes were prepared with deionized water obtained from a Millipore purification system (18.2 MΩ·cm resistivity, residual organic impurities at the low‐ppb level). Prior to use, all electrolyte solutions were purged with ultrapure argon. Before and during mounting the EC‐STM cell and filling it with electrolyte, the EC‐STM chamber was continuously flushed with Ar at 2 L min^−1^. During measurements, the chamber was kept under an Ar atmosphere maintained at 0.3–0.5 bar above ambient pressure. All chemicals were purchased from Sigma–Aldrich at the highest commercially available purity and used without further purification. STM tips were prepared by electrochemical etching of 0.25 mm tungsten wire in 2 M KOH, followed by rinsing with high‐purity water and drying. To suppress faradaic leakage currents and improve imaging stability, the etched tips were subsequently coated by passing them through a thin film of hot‐melt adhesive using an automated tip coater (ATC) [27]. An Au wire was used as the counter electrode, while a Mini‐HydroFlex reversible hydrogen electrode (RHE, Gaskatel) served as the reference electrode unless stated otherwise. Therefore, all electrode potentials (E) are reported vs RHE. To minimize ohmic drop, the reference electrode was positioned within 0.5 cm of the sample surface.

The tip bias potentials are reported with respect to the working electrode. Thus, the tip potential relative to the RHE reference electrode can be expressed as:

$$\;U_{b\;vs\;RHE}=E+U_{b}$$

where *U*_*b* *vs* *RHE*_ denotes the tip potential vs the reference electrode, *E* is the electrode potential, and *U_b_* is the bias applied between the tip and the working electrode.

STM image analysis was performed using WSxM [28] and LFA [29] software.

#### 4.2.2 XPS

Photoemission spectra were acquired in an ultrahigh‐vacuum (UHV) chamber with a working pressure below 5 × 10^−9^ mbar, using a non‐monochromatic Al Kα line (hν = 1486.7 eV) and an electron analyzer (VG Escalab MK II) set to a pass energy of 20 eV. The deconvolution into single chemical‐shift components was carried out using Voigt functions.

#### 4.2.3 STM

STM topographies were acquired in constant current mode, either under ambient conditions with Pt‐Ir tips, using either a Molecular Imaging or an Agilent Keysight 5500 instrument, or under UHV conditions using a SPECS SPM Aarhus 150 instrument. In general, for the acquisition of high‐resolution images, positive bias in the range between 0.5 and 1.0 V and tunneling current of 60–100 pA were used.

### 4.3 DFT Calculations

All density functional theory (DFT) calculations were performed using the Vienna Ab initio Simulation Package with the projector augmented‐wave (PAW) method and recommended PAW potentials [30, 31]. The Perdew–Burke–Ernzerhof (PBE) generalized gradient approximation was used for the exchange‐correlation functional, with Grimme's DFT‐D3 dispersion correction [32, 33] to account for van der Waals interactions. A plane‐wave kinetic energy cutoff of 400 eV was employed. Electronic states were occupied according to Gaussian smearing with a width of 0.1 eV. The Brillouin zone was sampled at the Gamma point only. Electronic self‐consistency was converged to 10^−5 ^eV, and ionic relaxations were performed using the conjugate‐gradient algorithm (IBRION = 2) until all forces were below 0.05 eV/A.

The Au(111) surface was modeled as a four‐layer slab in a (8 × 4) orthogonalized supercell (192 Au atoms) with a lattice of 23.6 × 20.4 A^2^ and ∼10 A of vacuum separating periodic images in the surface‐normal direction. The bottom three layers were fixed at their bulk positions, while the top layer and all adsorbate atoms were allowed to relax. NHC‐Au chains were constructed based on our previous work [7].

Hydrogen adsorption free energies were calculated following the standard approach of Norskov et al. [23]. Multiple adsorption sites and binding modes were sampled on each surface model.

## Funding

This research was funded by the National Science Center (Poland) under the SONATA 17 Project No. 2021/43/D/ST3/02873. We acknowledge financial support under the National Recovery and Resilience Plan (NRRP), Mission 4, Component 2, Investment 1.1, by the Italian Ministry of University and Research (MUR), funded by the European Union – NextGenerationEU– Project Title “Microscopic approach to Understand Synergies in Electrocatalysis” – Project No. 2022E5L4Y2 – CUP: C53D23003780006. We acknowledge support from Project C2 chemical complexity (CUP: C93C22009260001) under the MUR program “Dipartimenti di Eccellenza 2023–2027”. Financial support was provided by the US Department of Energy (DOE), Office of Science, Basic Energy Sciences (BES), Materials Sciences and Engineering Division under contract DE‐AC02‐05‐CH11231 (Nanomachine program KC1203) (synthesis of NHC precursors). B.Q. acknowledges support through a Bakar Institute of Digital Materials for the Planet Postdoctoral Fellowship. The University of Padova is acknowledged for financial support through the grant #01BIRD2022‐UNIPD (MUSYCA) and P‐DiSC#02BIRD2024UNIPD VS‐PpyTM. Z.Z. acknowledge the support from Stanford Energy Fellowship, Precourt Institute of Energy, and Stanford Chemical Engineering.

## Conflicts of Interest

The authors declare no conflicts of interest.

## Supporting information




**Supporting File 1**: smll75601‐sup‐0001‐SuppMat.docx.

**Supporting File 2**: smll75601‐sup‐0002‐MovieS1.docx.

## Data Availability

The data that support the findings of this study are available from the corresponding author upon reasonable request
