# Supplementary material for: A Dynamic Surface Metal‐Organic Framework Electrocatalyst: Potential‐Driven Activation of Atomically Defined Au Sites for the Hydrogen Evolution Reaction
Source: Small. 2026 Sep 12;22(57):e75601. doi: 10.1002/smll.75601 (PMC13659790; doi:10.1002/smll.75601)
Supplement: Supplementary file 1 [file SMLL-22-e75601-s002.docx]

Supporting Information

A Dynamic Surface Metal-Organic Framework Electrocatalyst: Potential-Driven Activation of Atomically Defined Au Sites for the Hydrogen Evolution Reaction

Paulina Wira^1ǂ^, Leonardo Cielo,^2ǂ^ Zisheng Zhang^3*^, Mattia Cattelan,^2*^ Rafał Lewandków^1^, Dennis Pedersoli^2^, Fujia Liu^4^, Boyu Qie^4,6^, Felix. R. Fischer^4,5,6,7^, Francesco Sedona^2^, Cristina Tubaro^2^, Tomasz Kosmala^1*^, and Stefano Agnoli^2^*

**Figure S1. (a)** STM image of the NHC based MOF prepared in vacuum conditions after the exposure to air for 2 days. The image was acquired in air at room temperature with Pt/Ir tip.
I_t_ = 80 pA, U_b_= 800 mV, size = 64.8 nm x 64.8 nm; **(b)** high resolution image showing the order withing the 1D chains, Au^δ+^ nodes are imaged as bright spots. I_t_ = 80 pA, U_b_ = 1000 mV,
size = 20.7 nm x 20.7 nm.

**Figure S2.** Consecutive *operando* EC-STM images of the SMOF/Au(111) interface recorded at a constant potential of −121 mV versus RHE in 0.1 M HClO_4_, corresponding to a current density of approximately −86 µA cm^−2^. Persistent molecular diffusion and reorganization
are observed under sustained HER conditions, demonstrating that the dynamic behavior
is not restricted to potentiodynamic measurements. Tunneling conditions: **(a-d)** U_b_ = -130 mV,
I_t_ = 1 nA***.***

**Figure S3.** Potential-dependent SMOF mobility and substrate restructuring. *Operando*
EC-STM images of a SMOF on Au(111) in 0.1 M HClO_4_ contrasting: **(a)** a highly mobile state of the framework at catalytic potentials, and **(b)** restricted mobility upon shifting to anodic potentials, allowing the molecular network to be clearly resolved**. (c)** Height profile
along the line in panel **(a)**, confirming the formation of a monolayer Au ad-islands.
Tunneling conditions: **(a)** U_b_ = 96 mV, I_t_ = 0.48 nA, E = -121 mV, *j* = -78.312 µA cm^-2^;
**(b)** U_b_ = -375 mV, I_t_ = 0.48 nA, E = 727 mV, *j* = 0.080 µA cm^-2^.

**Figure S4.** Time-resolved observation of potential-induced Au ad-island growth.
Sequential *operando* EC-STM images **(a–c)** show the morphological evolution of a freshly prepared liquid-phase SMOF/Au(111) interface in 0.1 M HClO_4_, capturing the formation
and growth of Au ad-islands. **(a)** Surface recorded in the double-layer regime.
**(b)** Image recorded after stepping the potential to −105 mV, corresponding to the HER onset and initiating of ad-island growth. **(c)** Image recorded after Δ*t* = 522 s at *E* = −105 mV.
**(d)** Height profile along line P1 in panel **(c)**, showing the topography of the original substrate step edge and multiple newly formed Au ad-islands. Tunneling conditions: **(a)** U_b_ = -117 mV, I_t_ = 1 nA, E = -26 mV, *j* = -2.5 µA cm^-2^; **(b)** U_b_ = -117 mV, I_t_ = 1 nA, E = -105 mV,
*j* = -4.0 µA cm^-2^; **(c)** U_b_ = -117 mV, I_t_ = 1 nA, E = -105 mV, *j* = -8.6 µA cm^-2^.

**Table S1.** Statistical analysis of Au ad-islands formed at the liquid-synthesized SMOF/Au(111) interface, based on the *operando* EC-STM images shown in **Figure S3**. Listed are the number of ad-islands, total island area, total island perimeter and area per island determined
for panels **(a–c)**, showing the progressive increase in Au ad-island population and size upon polarization to −105 mV.

| **Image** | **Number of ad-islands** | **Area (nm^2^)** | **Perimeter (nm)** | **Area per island (nm^2^)** |
| --- | --- | --- | --- | --- |
| Figure S4a | 7 | 107.1 | 122.2 | 15.3 |
| Figure S4b | 39 | 337.8 | 520.6 | 8.7 |
| Figure S4c | 68 | 932.4 | 1127.5 | 13.7 |

**Figure S5.** EC-STM images of the Au(111) surface recorded **(a)** in the double-layer
regime after several potential cycles toward the HER region, **(b)** under catalytic HER
conditions, and **(c)** after returning to the double-layer regime. No changes in the surface morphology are observed. Tunneling conditions: **(a)** U_b_ = -39 mV, I_t_ = 5.42 nA, E = -52 mV,
*j* = -0.5 µA cm^-2^; **(b)** U_b_ = -39 mV, I_t_ = 5.4 nA, E = -248 mV, *j* = -160.3 µA cm^-2^;
**(c)** U_b_ = -39 mV, I_t_ = 5.4 nA, E = -52 mV, *j* = -0.6 µA cm^-2^.

**Figure S6.** Au islands vacancies generated by SMOF restructuring. **(a-c)** EC-STM images
of a SMOF/Au(111) in 0.1 M HClO_4_ electrolyte. White circles highlight substrate vacancies formed by the extraction of gold atoms during the potential-induced dynamic activation
of the framework. Tunneling conditions: **(a)** U_b_ = -164 mV, I_t_ = 1 nA, E = 51 mV;
**(b)** U_b_ = -291 mV, I_t_ = 1 nA, E = 445 mV; **(c)** U_b_ = -316 mV, I_t_ = 1 nA, E = 1 mV.

**Figure S7**. Distinct terminal motifs in 1D SMOF chains. **(a-b)** High-resolution EC-STM images of a SMOF/Au(111) in 0.1 M HClO_4_. Green arrows pinpoint chain terminations
with an Au atom, whereas red arrows highlight terminal NHC molecular units.
Tunneling conditions: **(a)** U_b_ = -271 mV, I_t_ = 1 nA, E = 23 mV; **(b)** U_b_ = -315 mV, I_t_ = 1 nA,
E = -77 mV.

**Figure S8**. Long-term electrochemical stability and sustained HER catalytic performance
of SMOF/Au(111). **(a)** Selected CVs (cycles 1, 20, 40, 60 and 80) demonstrating a highly consistent catalytic response under continuous potential sweeping. **(b)** Comparative LSVs acquired on Day 1 (before the CV series shown in **(a)**) and Day 5 (after completion of the CV series), confirming the long-term durability of the system with no degradation in HER performance.

**Figure S9.** N 1 s XPS spectra, the bottom spectrum corresponds to the as-deposited molecules, while the top spectrum shows the sample after EC work. The deconvolution into single chemically shifted components is explained in the text.

**Figure S10.** DFT-optimized geometries of the pure Au sites with and without H*.

**Figure S11.** DFT-optimized geometries of the NHC-involved sites with and without H*.

**Figure S12**. First frame of **Supporting Movie S1**.

**Supplementary Note 1**

*Operando* potentiodynamic EC-STM visualization of potential-driven SMOF mobility
and catalytic activity. As the potential is scanned from the HER onset region into the active HER regime, the framework enters a highly dynamic state characterized by pronounced conformational fluxionality of the molecular arrays and rapid diffusion of Au nodes,
visible as characteristic scanning streaks and spikes. When the potential is subsequently reversed toward the double-layer regime, the molecular dynamics progressively slow down, revealing partial immobilization and spatial reorganization of the 1D arrays.
To directly correlate the structural evolution with the electrochemical response, the two graphs shown on the left display the electrode potential, E, and current density, *j*, versus time.
A red tracking dot continuously marks the electrochemical conditions corresponding
to each EC-STM frame.

Technical parameters. The movie contains 21 consecutive frames acquired over 726 s. Images were recorded in constant-current mode (I_t_ = 1 nA, bias indicated on-screen) and compiled
into a movie using WSxM software. The movie was exported at a playback speed of 10 fps.
